# The Role of Merkel Cell Polyomavirus and Other Human Polyomaviruses in Emerging Hallmarks of Cancer

**DOI:** 10.3390/v7041871

**Published:** 2015-04-10

**Authors:** Ugo Moens, Kashif Rasheed, Ibrahim Abdulsalam, Baldur Sveinbjørnsson

**Affiliations:** University of Tromsø, Faculty of Health Sciences, Institute of Medical Biology, NO-9037 Tromsø, Norway; E-Mails: kashif.rasheed@uit.no (K.R.); iab004@post.uit.no (I.A.); baldur.sveinbjornsson@uit.no (B.S.)

**Keywords:** polyomavirus, microRNA, inflammation, autophagy, glucose, therapy, exosomes

## Abstract

Polyomaviruses are non-enveloped, dsDNA viruses that are common in mammals, including humans. All polyomaviruses encode the large T-antigen and small t-antigen proteins that share conserved functional domains, comprising binding motifs for the tumor suppressors pRb and p53, and for protein phosphatase 2A, respectively. At present, 13 different human polyomaviruses are known, and for some of them their large T-antigen and small t-antigen have been shown to possess oncogenic properties in cell culture and animal models, while similar functions are assumed for the large T- and small t-antigen of other human polyomaviruses. However, so far the Merkel cell polyomavirus seems to be the only human polyomavirus associated with cancer. The large T- and small t-antigen exert their tumorigenic effects through classical hallmarks of cancer: inhibiting tumor suppressors, activating tumor promoters, preventing apoptosis, inducing angiogenesis and stimulating metastasis. This review elaborates on the putative roles of human polyomaviruses in some of the emerging hallmarks of cancer. The reciprocal interactions between human polyomaviruses and the immune system response are discussed, a plausible role of polyomavirus-encoded and polyomavirus-induced microRNA in cancer is described, and the effect of polyomaviruses on energy homeostasis and exosomes is explored. Therapeutic strategies against these emerging hallmarks of cancer are also suggested.

## 1. Introduction

Polyomaviruses are naked, circular double-stranded DNA viruses that infect birds and mammals, and recently the first fish-associated polyomavirus was described [1,2]. The genome of most polyomaviruses is approximately 5000 base-pairs and encodes regulatory proteins and structural proteins. The major regulatory proteins are the large tumor antigen (LT-ag) and the small tumor antigen (st-ag), while at least two structural proteins (VP1 and VP2) form the capsid. The regulatory proteins are expressed early during infection and participate in viral replication and viral transcription, while the structural proteins are expressed later in the infection cycle [3]. Many polyomaviruses encode additional regulatory and structural proteins (e.g., ALTO, VP3, VP4, agnoprotein) [4,5,6]. 

Studies with mice in the 1950s initiated by Ludwik Gross, and extended by Sarah Stewart and Bernice Eddy led to the identification of the first polyomavirus. They showed that a filtrate from a mouse leukaemia could cause multiple tumors in new-born mice and later it was demonstrated that these multiple tumors were virus-indeed. Hence the virus was referred to as polyomavirus from the Greek πολύσ for many and ωµα for tumors (reviewed in [7]). The first primate polyomavirus was isolated in 1960 [8]. This virus, Simian virus 40 (SV40), was shown to transform cells, including human cells, to induce tumors in animal models, and to be present in human cancers. The oncogenic potential of SV40 primarily depends on its LT-ag, which can bind the tumor suppressor proteins p53 and pRb, interfere with DNA repair, apoptosis, cellular transcription, protein degradation, telomerase activity, immune- and inflammatory responses, and stimulate angiogenesis and cell migration. SV40 st-ag can contribute to transformation by inactivating protein phosphatase 2A [9,10]. Besides SV40 and murine polyomavirus, other non-human polyomavirus such as hamster polyomavirus, lymphotropic polyomavirus, and simian agent 12 were shown to possess oncogenic properties in cell cultures or animal models [11,12,13]. However, the oncogenic role of these viruses in their natural host is unclear. In fact, only one mammalian polyomavirus seems to be firmly associated with cancer in its genuine host. Raccoon polyomavirus (RacPyV) was first identified in tumors of frontal lobes and olfactory tracts from raccoons. Ten out of 52 (19%) raccoons had brain tumors within the cranial portion of their frontal lobe(s), and all tumors contained RacPyV DNA, though not tissues from 20 unaffected animals. RacPyV genome was episomal in all tumors tested [14]. One case of hamster polyomavirus-induced lymphoma in a hamster outside of the laboratory environment has been described [15], while two novel mammalian polyomaviruses have been isolated from benign tumors. A polyomavirus was isolated from fibropapilloma on the tongue of a sea lion, and the complete genome of another polyomavirus was amplified in a biopsy from a fibroma on the trunk of an African elephant [16,17]. Further studies are required to assess whether these mammals are the genuine host, and whether these polyomaviruses are the causal infectious agent of such hyperplastic fibrous tissue in their natural host.

In contrast to mammalian polyomaviruses, bird polyomaviruses do not seem to induce tumors. Despite a similar genetic organization to that of mammalian polyomavirus, their LT-ag lacks homologies to the p53 binding sequences of mammalian polyomavirus and not all avian polyomavirus LT-ag possess the consensus sequence LXCXE required for pRb binding [18].

## 2. Human Polyomaviruses and Cancer 

The first two human polyomavirus viruses were isolated in 1971, and were named after the initials of the patient in which the virus was found: the BK virus (BKPyV) and the JC virus (JCPyV) [19,20]. Both BKPyV and JCPyV possess a genomic organization that resembles SV40 more than the murine polyomavirus. The former three viruses lack the middle T-antigen that is encoded by the murine polyomavirus, but have an additional late gene referred to as the agnogene [3]. Because the genomic organization of SV40 displays a higher functional and sequence similarity with the BKPyV and JCPyV, SV40 became the polyomavirus model system for unveiling the oncogenic mechanisms of this family [21,22]. Since 2007, 11 novel human polyomaviruses have been described: KIPyV, WUPyV, Merkel cell PyV (MCPyV), HPyV6, HPyV7, *Trichodysplasia spinulosa*-associated PyV (TSPyV), HPyV9, HPyV10 (and the isolates MW and MX), STLPyV, HPyV12, and NJPyV-2013 [23,24,25,26,27,28,29,30,31,32,33,34,35]. The seroprevalence of the different human polyomavirus ranges from ~25% to ~100% depending on the virus. The high seropositivity therefore demonstrates that these viruses are common in the adult human population [36,37,38]. 

Whereas the oncogenic properties of BKPyV, JCPyV and MCPyV in cell culture and animal models are well-documented [39,40,41,42], only MCPyV seems to be associated with cancer in its natural host. Approximately 80% of Merkel cell carcinoma tumors are positive for the MCPyV genome, which is typically integrated and encodes a truncated form of LT-ag [43]. BKPyV and JCPyV DNA, RNA and proteins have been detected in several tumor tissues, but are also often present in control non-malignant tissues [44,45,46]. Hence, a causal role for these viruses in human cancers remains controversial, although the presence of BKPyV may increase the risk of the development of renal and prostate cancer, while JCPyV may be associated with colorectal cancer and CNS tumors [47,48,49,50]. Polyomavirus-associated colorectal cancer may be due to other polyomaviruses present in meat as suggested by Harald zur Hausen [51]. Recent analyses of beef samples have identified several bovine polyomaviruses related to the human polyomaviruses MCPyV, HPyV 6, HPyV7 or other animal polyomaviruses including fruit bat polyomavirus, RacPyV and chimpanzee polyomavirus [52,53]. It remains to be established whether these viruses can be detected in human colorectal biopsies. The possible association of the other human polyomaviruses with cancer has been scarcely examined, and in only few cases was viral DNA or protein detected in tumor tissue (Table 1). Based on our present knowledge, convincing proof of their role in these cancers is lacking.

**Table 1:** Prevalence of the novel human polyomaviruses in human cancers. BK virus (BKPyV), JC virus (JCPyV), Merkel cell PyV (MCPyV) are not included.

	Number of samples	Method	Number of positive samples	Comments	Reference
Melanoma (st-age IV)	18	PCR and IHC (HPyV6 VP1moAb)	HPyV6: 18 HPyV7: 17 TSPyV: 4 HPyV9: 1 HPyV10: 12	Low viral DNA loads, but higher for HPyV6	[54]
Mucosal melanoma	37	PCR	KIPyV: 0 WUPyV: 0 HPyV6:0 HPyV7:0 TSPyV: 0 HPyV9:0 MWPyV: 0		[55]
Squamous cell carcinoma	63	PCR	HPyV6: 2 HPyV7: 1	Low viral DNA loads	[56]
Basal cell carcinoma	50	PCR	HPyV6: 1 HPyV7: 2	Low viral DNA loads	[56]
Melanoma	47	PCR	HPyV6: 2 HPyV7: 2	Low viral DNA loads	[56]
Basal cell carcinoma	41	PCR	HPyV6:3 HPyV7:0 TSPyV: 0 HPyV9:0		[57]
Squamous cell carcinoma	52	PCR	HPyV6:2 HPyV7:0 TSPyV: 0 HPyV9:0		[57]
SCC *in situ*	8	PCR	HPyV6:1 HPyV7:0 TSPyV: 0 HPyV9:0		[57]
Keratoacanthoma	42	PCR	HPyV6:2 HPyV7:0 TSPyV: 0 HPyV9:0		[57]
Microcystic adnexal carcinoma	5	PCR	HPyV6:0 HPyV7:0 TSPyV: 0 HPyV9:0		[57]
Atypical fibroxanthoma	14	PCR	HPyV6:0 HPyV7:0 TSPyV: 0 HPyV9:0		[57]
Actinic keratosis	31	PCR	HPyV6:1 HPyV7:0 TSPyV: 0 HPyV9:0		[57]
Breast cancer	54	PCR	HPyV6: 1 HPyV7:1		[58]
Merkel cell carcinoma		deep sequencing	HPyV6: 1 HPyV7:1 HPyV9:1		[59]
Extracutaneous melanoma	38	PCR	KIPyV: 0 WUPyV: 0		[60]
SCC+AK	142	deep sequencing	HPyV6: 1		[61]
Chronic lymphocytic leukaemia	27	PCR	HPyV9: 0		[62]
Primary cutaneous B-cell lymphomas (CBCLs) or cutaneous T-cell lymphomas (CTCLs)	130	PCR	HPyV6: 6 HPyV7: 1 TSPyV: 0		[63]
MCC	28	PCR	HPyV6: 0 HPyV7:0		[64]
Pilomatricomas (benign skin tumor associated with hair follicles	?	?	TSPyV: 0		[65]
Lung cancer	20	PCR	KIPyV:9		[66]
CNS tumors	25	PCR	KIPyV: 0 WUPyV: 0		[67]
Neuroblastoma	31	PCR	KIPyV: 0 WUPyV: 0		[67]
Acute lymphoblastic leukaemia	50	PCR	KIPyV: 0 WUPyV: 0		[68]
Lung cancer	30 32	PCR PCR	KIPyV: 0 WUPyV: 0 KIPyV: 0 WUPyV: 0		[69] [70]
Neuroendocrine tumors	50	PCR	KIPyV: 0 WUPyV: 0 HPyV6:0 HPyV7:0 TSPyV: 0		[71]
Skin lesions from CTCL patients	39	PCR	HPyV6:11 HPyV7:5 TSPyV: 0 HPyV9:0		[72]
Blood from CTCL patients	39	PCR	HPyV6:0 HPyV7:0 TSPyV: 0 HPyV9:0		[72]
Glioblastoma multiforme	39	PCR	HPyV6:0 HPyV7:0 HPyV9:0		[73]
Thymic epithelial tumors Thymic hyperplasias Foetal thymus tissue	37 20 20	PCR, FISH, IHC	PCR FISH IHC HPyV7: 20 23 17 HPyV6: 0 HPyV7: 8 14 6 HPyV7: 0		[74]

**Figure 1:**
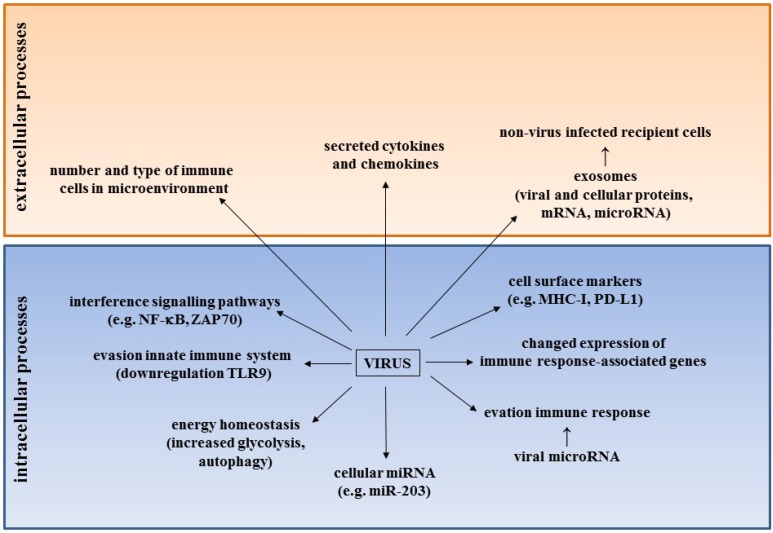
Novel mechanisms by which HPyV may contribute to cancer. See text and Table 2 for details.

The cancer biology of BKPyV, JCPyV and MCPyV has been extensively reviewed by others [39,43,45,46,75,76,77] and is also discussed by others in this special issue on Tumor Viruses. This review will focus on novel strategies that human polyomaviruses may use to transform cells. Figure 1 summarizes the novel mechanisms by which HPyV may contribute to cancer. 

## 3. HPyV and Emerging Hallmarks of Cancer

### 3.1. The Immune System and HPyV in Cancer

Individuals with a dysfunctional immune system are more disposed to diseases, infections and (viral-induced) cancers. Moreover, oncoviruses can induce inflammation, which may predispose host cells to acquire carcinogenic mutations [78]. In accordance with the cancer immunoediting hypothesis, tumor cells need to proficiently traverse separate phases in a sequential order to attain cancer manifestation and progression. These phases constitute interactions between the immune system and the cancer cell, and include the elimination of newly transformed cells, an equilibrium in which the immune system restrains the outgrowth of tumors, and an escape in which the tumor cells are able to circumvent the host immune response phases [79,80,81]. For a virus to induce tumors, they need to circumvent elimination by the immune system and to induce alternations in the tumor microenvironment, including in the infected cell allowing the virus-transformed cell to progress [82,83]. Because MCPyV is the only HPyV associated with cancer, the main focus will be on MCPyV’s interaction with the immune system. 

Epidemiologic data show that patients with T cell dysfunction are at a 5- to 50-fold increased risk of developing MCC, thereby indicating the importance of the immune system (reviewed in [83]). However, immunocompetent individuals may also develop MCPyV-positive MCC, suggesting that the virus and virus-infected cells can avoid elimination by the immune system. 

#### 3.1.1. HPyV and Evasion of the Innate Immune System

One mechanism by which MCPyV circumvents the immune system is to abate the innate defence mechanism. MCPyV LT-ag and st-ag downregulate the Toll-like receptor 9 (TLR9), an important receptor of the host innate immune system that senses viral dsDNA in epithelial and MCC cells [84]. LT-ag inhibits TLR9 expression by decreasing the mRNA levels of the transcription factor C/EBPβ. LT-ag of BKPyV, but not JCPyV, KIPyV and WUPyV, is also able to repress TLR9 expression. Interestingly, C/EBPβ has a vital role in regulating IL-6, IL-8, and TNF-α cytokine transcription [85]. Moreover, it is also suggested that C/EBPβ has a tumor-suppressive activity by down-regulating CDK2, CDK4, and E2F complex activity [86,87]. Thus MCPyV LT-ag mediated suppression of C/EBPβ expression may perturb immune responses and provoke cell proliferation.

#### 3.1.2. Immune Cells in the Microenvironment of MCC

To investigate inflammatory modulators in MCC required for escaping of the tumor from immune surveillance, and to deduce a possible contribution of MCPyV in oncogenesis, several groups have examined immune cells and inflammatory mediators virus-positive and virus-negative MCC. Differences in immune and inflammatory cells, markers, and gene expression in MCPyV-positive and MCPyV-negative MCC tumors are summarized in Table 2. Compared to virus-negative tumors, a higher number of infiltrating CD8+ T-cells in MCPyV-positive MCC has been observed [89,90,91], while others group have not detected a relationship with virus status and the number of intratumoral CD8+ T-cells [92,93]. Other differences in the microenvironment of virus-positive and virus-negative MCC include a higher number of CD3+ T-cells, CD20+ B cells, CD16+ natural killer cells, and CD68+, CD69+, CD163+ macrophages [88,89,90,93,94,95]. FoxP3+ regulatory T-cells were present in 4/4 LT-ag positive MCC, whereas 3/6 LT-ag negative tumors did not contain FoxP3+ regulatory T-cells [93].

**Table 2:** Immune cells and inflammatory mediators in MCPyV-positive and MCPyV-negative Merkel cell carcinoma (MCC).

Component	MCPyV-positive *versus* MCPyV-negative MCC	Reference
**Cells in tumor microenvironment**		
-CD3+ T-cells		
	higher number in MCPyV-positive MCC	[88,89,90]
-CD4+ T-cells		
	high number associated with high LT-ag expression	[90]
-CD8+ T-cells	higher number in MCPyV-positive MCC	
		[89,91,92]
- CD16+ natural killer cell	higher number in MCPyV-positive MCC	
		[88,90]
-CD20+ B cells	more common in MCPyV-positive MCC;	
	no significant difference between MCPyV-positive and **–**negative MCC	[93] [89]
-CD68+ macrophages	higher number in MCPyV-positive MCC	
		[88,90,94,95]
-CD69+ macrophages	higher number in MCPyV-positive MCC	
		[90,94,95]
-FoxP3+ regulatory T-cells	more common in MCPyV-positive MCC	
		[93]
**Cell surface markers:**		
-CD3D	enrichment of transcripts in MCPyV-positive MCC	[89]
	enrichment of transcripts in MCPyV-positive MCC	
-CD3G	lacking in CD8+ T-cells	[89]
		
-CXCR3	lower levels in MCPyV-positive MCC	[93]
		
-MHC-I	higher in MCPyV-positive MCC	[96]
		
-PD1	higher in MCPyV-positive MCC	[95,97,98]
		
-Tim-3		[97]
**Signal transduction proteins**		
-NFκB levels	lower in MCPyV-positive MCC	[99]
		
-IκB levels	lower in MCPyV-positive MCC	[99]
		
-TANK		
	reduction in MCPyV st-ag expressing cells MCC13 cells	[99]
- ZAP70	compared to virus-negative cells	
	enrichment of transcripts in MCPyV-positive MCC	[89]
**Cytokines/chemokines**		
-CCL20	reduction in MCPyV st-ag expressing cells MCC13 cells	[99]
	compared to virus-negative cells	
-CXCL-9	reduction in MCPyV st-ag expressing cells MCC13 cells	[99]
	compared to virus-negative cells	
-IL-2	reduction in MCPyV st-ag expressing cells MCC13 cells	[99]
	compared to virus-negative cells	
-IL-8	reduction in MCPyV st-ag expressing cells MCC13 cells	[99]
	compared to virus-negative cells	
-Prokineticin 1 mRNA	higher in MCPyV-negative MCC	[90]
		
-Prokineticin 2 mRNA	higher in MCPyV-positive MCC	[90]
**Other differentially expressed proteins**		
-granzyme B (role in apoptosis)		
	Expression was rare in CD8+ cells	[93]

Afanasieve *et al*. proposed that MCC tumors may prevent the invasion of lymphocytes by a reduction of E-selectin-positive vessels within the tumors because the downregulation of E-selectin in human squamous cell carcinomas was associated with a restricted entry of T-cells into tumors [100,101]. Of 56 tested MCC biopsies, approximately half displayed a reduction of E-selectin-positive vessels within the tumors compared with vessels in peritumoral areas [102]. However, the association between the presence of virus and E-selectin levels was not investigated.

#### 3.1.3. Changes in Expression of Cell Surface Markers on MCC Cells

Expression of cell surface markers was performed to determine the functionality of the immune cells. These analyses revealed that the expression of MHC-I in MCPyV-positive MCC was significantly lower than in virus-negative MCC [96]. Cell-surface MHC-I expression was down-regulated in 84% (n = 114) of MCC, and approximately half of the tumors had poor or undetectable MHC-I levels. The downregulation of MHC-I expression has been identified as a vital immune evasion strategy used by several viruses, including oncoviruses [103,104,105,106,107,108]. An identical mechanism can be employed by MCPyV, but it remains to be determined as to whether viral proteins are implicated in MHC-I down-regulation. Tumors that undergo a significant downregulation of MHC-I should become a target of natural killer cells. MCC can avoid this by e.g., reducing the expression of NK-activating receptors such as natural killer group 2, member D (NKG2D) [109]. Interestingly, BKPyV and JCPyV microRNA target ULBP3, which is the ligand of NKG2D (see further), though it is not known whether MCPyV microRNA targets ULBP3 or NKG2D. Another surface marker that was differentially expressed on MCPyV-positive and negative tumors is the immune-inhibitory ligand programmed death ligand-1 (PD-L1) [95,97,98]. The major receptor for PD-L1, PD-1 is expressed by activated T lymphocytes, and when this receptor is engaged by its ligands PD-L1 it serves to inhibit the T-cell response. PD-L1 may be aberrantly expressed by tumor cells and protect against immune attack [110]. The number of intra-tumor T-cells is commonly higher in virus-positive MCC than virus-negative MCC, and PD-1 was expressed on a high percentage of MCPyV-positive tumors [95,97,98]. Moreover, approximately 50% of MCPyV-positive MCC express PD-L1 on tumor cells, while no expression was detected in MCPyV-negative MCC. Hence, the association between PD-1-positive cells and PD-L1 expression in the tumor microenvironment seems to create immune resistance by the tumor, thereby allowing the tumor to progress [97,98]. The mechanism by which MCPyV provokes the expression of PD-L1 remains to be determined, but Lipson and co-workers anticipated that IFN-γ may drive PD-L1 expression, but other interleukins such as IL-6, IL-10, IL-17 and IL-21 cannot be excluded. A role for PD-1 positive cells in protecting PD-L1-expressing MCC cells is buttressed by observations in a complete or partial regression of MCC. The exact mechanism for spontaneous regression is not known, although T-cell-mediated response and apoptosis by T-cells has been suggested [111]. The rate of regression of MCPyV-positive *versus* MCPyV-negative MCC has not been evaluated, but complete regression has been reported in a 76-year old Japanese man with virus-positive MCC [112]. In this patient, only ~3% of the tumor-infiltrating T-cells were PD-1 positive, while in three other patients (females, mean age 81.3 years) with MCPyV-positive MCC who did not show any regression of the tumor, 18.2%–23.0% of the T-cells were of PD-1 positive. This suggests that a reduction of PD-1-positive T-cells may be associated with spontaneous tumor regression [112]. Another surface protein that was aberrantly expressed on immune cells in the tumor microenvironment was CXCR3 [97]. All CD8+ cells lacked CXCR3, thus indicating that these T-cells were functionally compromised. CXCL12 or stromal cell-derived factor 1, a chemokine with pleiotropic functions, including the attraction of inflammatory cells [113], was expressed outside malignant nodules, but its receptor CXCR4 was expressed by tumor cells, though not on infiltrating CD8+ cells. Finally, the cell-surface protein T-cell immunoglobulin and mucin domain-3 (Tim-3), which also functions to inhibit T-cell responses, was also upregulated on infiltrating T-cells in MCPyV-positive MCC [97].

#### 3.1.4. Expression Profile of Genes Associated with the Immune Response in MCC

Gene expression profile analysis has been applied to identify differentially expressed genes in MCPyV-positive and MCPyV-negative MCC. Microarray technology, using >54,000 probes, identified 1593 genes that were differently (≥2-fold) expressed comparing virus-positive and virus-negative MCCs [89]. An enrichment of genes associated with the immune response included genes encoding the δ and γ chains of CD3, the tyrosine kinase ZAP70, which plays an important role in the T-cell response, and the C-region of the µ heavy chain. Another approach compared the transcriptome from cells with an inducible expression of MCPyV st-ag with that of control cells, and revealed that the induction of st-ag expression resulted in ≥2-fold reduced transcript levγels of genes associated with the immune response such as CCL20, CXCL-9, IL-2, IL-8 and TANK, a negative regulator of TLR signaling. Less CCL20 and IL-8 were secreted by MCC13 cells expressing MCPyV st-ag compared with virus-negative MCC13 cells after TNFα stimulation [99]. mRNA profiling of 35 MCC tumors (both MCPyV-positive and negative) with favorable prognoses overexpressed genes such as components of cytotoxic granules (granzymes A, B, H and K), chemokine CCL19 and chemokine receptor 2, MHC-II and NKG2D [92]. The contribution of MCPyV on the expression of these genes cannot be appreciated because the data originate from both MCPyV-positive and MCPyV-negative tumors. Gene expression profiling of MCC tumor cells showed the lack of expression of IL-2 and IFN-γ, whereas IL-12 was expressed [113]. However, this study was performed before MCPyV was identified, so therefore a role of the virus in altered gene expression cannot be deduced. Another study monitored the transcript levels of the chemokine-like proteins prokineticin-1 and prokineticin-2, which are involved in angiogenesis, inflammation and cancer. MCPyV-positive MCCs had a higher than median prokineticin-2 mRNA levels, while virus-negative tumors had a higher than median prokineticin-1 transcript levels [90]. A high tumor prokineticin-2 mRNA content was associated with the expression of MCPyV LT-ag. The biological relevance of this observation for virus-induced MCC remains to be established. Wheat and co-workers observed that the expression of granzyme B, a mediator of apoptosis [114], was rare in MCC infiltrating CD8+ cells, hence suggesting that these cytotoxic T cells were functionally compromised [93].

#### 3.1.5. Effect of st-ag on the NF-κB Pathway

The molecular mechanism by which MCPyV may perturb gene expression in virus-positive MCC tumor cells is not known, but several of the genes listed in Table 2 (e.g., CXCL9, IL-2, IL-8, MHC-I, IκB) are known to be a target for NF-κB [115,116]. Interestingly, MCPyV st-ag was shown to downregulate NF-κB-mediated transcription [99]. St-ag-mediated inhibition of the NF-κB pathway seems to require an interaction of st-ag with NF-κB essential modulator (NEMO) adaptor protein and protein phosphatases 2A and 4C. This will prevent IKKα/IIKβ-mediated phosphorylation of IκB, thus leading to a reduced nuclear translocation of NF-κB. MCPyV interference with the NF-κB pathway is further sustained by the observations that IκB levels were 60% lower in the MCPyV-positive MCC cell line MKL-1 compared with MCPyV-negative MCC13 cells, and by a declined expression of NF-κB and NF-κB-associated genes in virus-positive MCC compared to virus-negative MCC [99,117]. All these findings indicate that MCPyV interferes with the NF-κB pathway, and that MCPyV st-ag may help the virus to evade the host antiviral defence and to persist in the infected cell [99]. It is not known whether the st-ag of other HPyV has the same property, but residues 95 to 111, which are crucial for the interaction between MCPyV st-ag, NEMO and PP2A and PP4C are not conserved [118]. Interestingly, ultraviolet (UV) exposure, a risk factor for MCC [119], was shown to stimulate mutations in LT-ag and increase the expression of st-ag in the tumor cells [120]. Hence, UV exposure may be a virus-dependent mechanism that promotes MCPyV-induced MCC through the aforementioned st-ag:NF-κB interaction. 

#### 3.1.6. Viral Microrna and Evation of the Immune Response 

Another mechanism by which HPyV may affect gene expression is by microRNA. MicroRNAs (miRNAs) are small RNAs that can down-regulate protein production by either degrading transcripts or inhibiting the translation of mRNA. SV40 miRNA, the first PyV miRNA to be described, was shown to reduce cytotoxic T lymphocyte-mediated lysis and IFN-γ release [121], whereas other HPyV seem to apply different strategies to escape the immune system. BKPyV, JCPyV and MCPyV miRNA were unable to inhibit IFN-induced transcription of the luciferase reporter gene [122], but BKPyV and JCPyV miRNAs inhibited the translation of UL16-binding protein 3 (ULBP3) mRNA [123]. ULBP3 is a ligand recognized by natural killer group 2, member D (NKG2D) receptor. NKG2D is expressed by NK and CD8+ T-cells and binding to ULBP3 triggers killing of the target cell [124]. Consequently, BKPyV- and JCPyV-infected cells may escape from NKG2D-mediated killing and circumvent the immune system. The proteins PSME3 and PIK3CD/p110δ, which are implicated in immune functions, were predicted to be putative targets for MCPyV miRNA [125]. PSME3 is a subunit of a proteasome responsible for the generation of peptides loaded onto MHC I, and PI3KCD plays a unique role in antigen receptor signaling by activating T-cells and B-cell proliferation [126,127,128]. The depletion of these proteins may prevent MCPyV infection to be cleared by the immune system, thereby allowing the viral infection to sustain. One of the SV40 strain RI257 miRNA targets is α-actinin 4 (ACTN4), a protein that activates the NFκB pathway [129]. Stable knockdown of ACTN4 reduces TNFα-mediated induction of NFκB and expression of e.g., IL-1β [130]. SV40-RI257I miRNA may therefore interfere with inflammatory responses. The 3p, and the 5p miRNAs of BKPyV and JCPyV share sequence identity (16 out of 22 nucleotides) with SV40-RI257I miRNA [6], but it is not known whether they also target ACTN4. SV40 strain 776 microRNA was shown to diminish the expression of the Serine/Threonine kinase MST4 in the African green monkey kidney epithelial cell line BSC-40, though not in human embryonal kidney 293T cells [129]. Interestingly, knockdown of MST4 in mice resulted in an exacerbated inflammation upon septic shock [131]. It is not known whether any of the HPyV encodes a miRNA that targets MST4, but if so, the following scenario can be imagined: A persistent HPyV infection may result in the depletion of MST4, thus causing the aggravation of inflammatory responses and a contribution to malignancy.

### 3.2. The Role of HPyV microRNA and HPyV-induced microRNA in Cancer

Some polyomaviruses have been shown to express viral miRNA, while others may encode a putative miRNA [6,132,133,134]. Although several viral miRNAs have been suggested to play a role in cancer [135], a direct implication of HPyV miRNA in cancer is lacking. Because RacPyV and MCPyV are the only PyV to so far be associated with cancer in their natural host, the expression of their miRNAs was examined in tumors. RacPyV miRNA was among the most abundant miRNAs detectable in RacPyV-associated tumors, but was not observed in RacPyV-negative non-tumor raccoon tissue [134]. This stands in contrast to MCPyV-positive MCC tumors, in which viral miRNA is only detectable in less than half of the tumors tested, and when present, MCPyV miRNA levels were <0.025% of total miRNAs in MCPyV-positive MCC [125,136]. This observation suggests that MCPyV miRNA is not involved in MCC.

PyV miRNA can modulate biological activities that can contribute to malignancy such as evading the immune system, apoptosis and perturbing cellular gene expression [137,138]. The role of HPyV-encoded miRNA in immune evasion was discussed above. PyV miRNAs may also prevent apoptosis. MCPyV miRNA targets the host cell protein AMBRA1, which is involved in autophagy and apoptosis [139], while mouse PyV miRNA downregulates the pro-apoptotic factor Smad2, resulting in a suppression of apoptosis *in vivo* [140]. Aberrant cellular gene expression may promote neoplastic progression, and PyV miRNAs may perturb cellular gene expression by interfering with splicing, thereby targeting transcription factors or proteins controlling the activity of transcription factors, or by inducing the expression of cellular miRNAs. SV40 miRNA is predicted to target the dual-specificity protein phosphatase DUSP8, a negative regulator of the JNK and p38 mitogen-activated protein kinases, whereas MCPyV may downregulate the expression of transcription factor RUNX1, the splicing factor RBM9/FOX2, as well as the repressor MECP2 [125,129]. Viral infection can induce a unique signature of host cell miRNAs, which may contribute to viral pathogenic processes [141]. MiRNAs are initially transcribed by RNA polymerase II, and SV40 LT-ag has been shown to interfere with RNA polymerase II-dependent transcription [142,143]. Hence, PyV infection may alter the pattern of cellular miRNA expression. However, a common feature shared by all known PyV miRNA is the silencing expression of LT-ag so that no effect on cellular miRNA expression is expected [121,122,132,134,144,145]. On the other hand, interference with cellular miRNA expression is plausible in MCPyV-positive tumors because MCC do express LT-ag [146], and RacPyV-positive tumors also express LT-ag [147]. The effect of LT-ag on cellular miRNA expression has not been investigated, but the proteins of the oncovirus HBV, EBV, KSHV and HCV help regulate the levels of cellular miRNAs, including oncogenic miRNAs [148,149,150,151,152,153,154]. 

Xie and co-workers compared miRNA profile in MCPyV-positive and negative MCC. One miRNA that was significantly lower expressed in MCPyV-positive MCC compared to MCPyV-negative MCC was miR-203. The overexpression of miR-203 in MCPyV-negative MCC inhibited cell growth and induced cell cycle arrest [155]. This finding suggests that MCPyV may cause cell proliferation by repressing the expression of miR-203, but the exact mechanism by which MCPyV may regulate this miRNA remains to be elucidated. 

### 3.3. Effect of HPyV on Energy Homeostasis

In healthy cells, glycolysis initiates in the cytoplasm where glucose is metabolized into pyruvate, which then enters the mitochondria where it is converted into acetyl-CoA and enters the Krebs’ cycle to generate ATP. In cancer cells, a metabolic switch occurs: the suppression of mitochondrial glucose oxidation and the upregulation of aerobic breakdown of glucose. This phenomenon was first described by Otto Heinrich Warburg, and is known as the Warburg effect [156]. While mitochondrial glucose oxidation generates 36 molecules of ATP per molecule of glucose, only two molecules of ATP are produced per molecule of glucose by aerobic breakdown. Cancer cells compensate for this by increasing the uptake of glucose and by stimulating the transcription of almost all the glycolytic enzymes in the cytoplasm [79,157,158]. Metabolism in Merkel cells and (MCPyV-positive) MCC has not been studied, but a Positron Emission Tomography (PET) scan of MCC with glucose analogues suggests a high rate of glycolysis in these tumors [159]. However, a possible role for MCPyV in enhanced glucose metabolism in MCC remains to be determined. Several studies with polyomavirus-transformed cells indicate that these viruses may affect glucose metabolism. Additionally, a redistribution of membrane glucose transporters, increased aerobic glycolysis and an increased activity of glycolytic enzymes were observed in SV40-transformed cells compared to non-transformed cells [160,161,162]. A role for JCPyV LT-ag in regulating the metabolic utilization of glucose in brain tumors has been recently suggested [163]. Another study showed that JCPyV LT-ag expressing medulloblastoma cells had a significantly lower mitochondrial respiration and glycolysis, but a three-fold higher consumption of glutamine compared to non-LT-ag expressing cells [164]. Oxygen consumption and glucose uptake were compared in fibroblast transduced with the telomerase catalytic subunit, or in combination with SV40 LT-ag or LT-ag plus st-ag. A progressive increase in both metabolic markers was measured, as cell lines expressed more oncogenes. This observation underscores a role for LT-ag and st-ag in decreasing the cell’s dependence on mitochondrial energy production [165]. SV40 st-ag can activate Akt, while Akt can stimulate the expression of glycolytic enzymes and aerobic glycolysis [166,167,168]. SV40 st-ag can also activate the MEK/ERK mitogen-activated protein kinase pathway, which can then increase glucose transport [169,170]. These findings suggest that st-ag may stimulate glucose uptake and aerobic glycolysis. The tumor suppressor p53 acts as an anti-Warburg molecule because it acts as a potent inhibitor of glycolysis [171]. The LT-ag of BKPyV and JCPyV has been shown to bind and inactivate p53 [172,173]. Although the LT-ag-mediated inactivation of p53 may promote glucose uptake and stimulate the glycolytic pathway, it may not be operational in MCPyV-positive MCC because the truncated form of LT-ag expressed in Merkel cell carcinomas does not bind p53 [40]. The interaction between p53 and LT-ag of other HPyV has not been investigated, but they all encompass a putative p53-binding motif [6].

Autophagy is another mechanism that allows cancer cells to maintain the levels of nutrients and energy in nutrient-limited environments, which helps to facilitate the survival of tumor cells. Moreover, autophagy regulates cellular invasion and metastasis [173]. The human tumor viruses EBV, KSHV, HBV, and HCV can modulate the autophagy pathway to favor viral infection by enhancing viral replication, prevent apoptosis or maintain a persistent and life-long infection [174]. Hence, viral interference with the autophagy pathway may contribute to tumorigenesis by these viruses, though less is known about the effect of HPyV on autophagy and the biological consequences. Bouley *et al*. could establish a supporting role for autophagy in BKPyV infection [175]. Using transformed human foreskin fibroblasts and HEK cells expressing or lacking the SV40 st-ag, it was demonstrated that st-ag helps to maintain energy homeostasis in glucose-deprivation cancer cells by activating AMP-activated protein kinase (AMPK), thereby inhibiting the mammalian target of rapamycin (mTOR) to shut down protein translation, and inducing autophagy as an alternate energy source. This protective role of st-ag under conditions of glucose deprivation depends on its ability to interact with protein phosphatase 2A [176]. It is not known whether the st-ag of other HPyV may exert similar functions, but BKPyV, JCPyV, MCPyV and MWPyV (HPyV10) st-ag have also been shown to interact with PP2A [99,177,178,179,180,181]. Other effects of HPyV on autophagy came from a study by Khalili *et al*., who found that JCPyV LT-ag suppressed the expression of Bcl-2-associated athanogene Bag3, a protein implicated in apoptosis and autophagy [182]. On the other hand, overexpression of Bag3 induces autophagy-mediated degradation of JCPyV LT-ag. Bag3 interacts with the C-terminal half region of LT-ag which encompasses a zinc finger structure and partially overlaps with the p53 binding domain [183]. Consequently, LT-ag may repress the expression of Bag3 and protect itself from being degraded by Bag3-mediated autophagy. MCPyV-positive MCCs express C-terminal truncated LT-ag, which may impede interaction with Bag3. Under stress conditions, primary neuroglial cells immortalized with SV40 LT-ag had increased levels of the autophagy marker LC3B compared to non-LT-ag expressing cells [184]. However, the biological implication was not investigated.

### 3.4. HPyV and Exosomes 

Exosomes are endosome-derived membrane vesicles of approximately 50 nm–100 nm in diameter that are shed by cells and act as a communication tool between cells. Exosomes contain cellular proteins, carbohydrates, lipids, DNA, rRNA, mRNA, siRNA and other non-coding RNAs (for recent reviews, see [185,186,187]). Exosomes secreted by tumor cells participate in the modulation of angiogenesis, cell proliferation, cell invasion, gene regulation and immune evasion, thereby creating advantages for malignant growth [186]. Exosomes released by virus-infected cells can also contain viral-derived components and are implicated in the pathogenesis of viruses. Indeed, the human oncoviruses Epstein-Barr virus, Kaposi sarcoma-associated herpes virus, hepatitis B virus, hepatitis C virus and human T-lymphotropic virus type 1 all utilize exosomes to transfer viral (onco)proteins, mRNA and miRNAs to non-infected cells [188,189,190,191,192,193,194]. Exosomes captured by target cells may facilitate the spread of (onco)viral proteins and nucleic acids, thereby promoting malignancy in the recipient cells in the absence of an infection by virions. Exosomes can provoke immune alterations that may play a role to create an immunotolerogenic microenvironment during the carcinogenesis process. They can promote host immune and inflammatory responses by activating T- and B-cells, and by releasing exosome-trapped inflammatory molecules such as TNFα and IL1β in the recipient cells. Even so, exosomes have also been shown to inhibit immune responses by preventing CD4^+^ T-cell proliferation, CD8^+^ CTL response or transporting anti-inflammatory molecules (reviewed in [187]).

The generation of exosomes by HPyV-infected cells has scarcely been investigated. Studies with mouse primitive glioblastoma-like brain tumor cell lines harbouring integrated SV40 large T-antigen DNA revealed the presence of SV40 large T-antigen sequences in exosomes produced by these cells [195]. Recently, JCPyV microRNA was detected in exosomes derived from human plasma and urine [196], although studies on the possible roles of exosomes released by HPyV-infected hosts cells are lacking.

## 4. Therapeutic Strategies against Emerging Hallmarks of Cancer

Specific inhibitors against HPyV are lacking, and the development of vaccines and vaccination are still in a very preliminary phase [197,198,199,200,201]. Therapeutic strategies directed against emerging features of cancer such as inflammation, immune evasion, exosomes, microRNA and energy homeostasis may offer alternatives to help combat HPyV-positive tumors. *In vitro* studies and MCC xenograft mouse models suggested a beneficial effect of IFN. Intratumoral administering of a mixture of different IFNα subtypes and IFNβ resulted in a regression of MCPyV-positive, but not MCPyV-negative xenografts of MCC cells, while IFNα-2b, IFNβ-1b, and IFNγ-1b challenge resulted in an increased cell-surface expression of MHC-I on MCC cell lines [96,202]. However, studies in patients with MCPyV-positive MCC have been proven to exhibit variable effects. Subcutaneous administration of IFN-β resulted in a complete regression of MCPyV-positive MCC tumors in a Japanese patient, but IFN-α-2b treatment of an 84-year-old man and an 81-year-old woman had no effect [203,204]. IFNβ stimulated MHC-I expression on tumor cells of 3/3 MCPyV-positive MCC patients [96]. Intralesional treatment of a 67-year old MCPyV-positive MCC patient with INFβ-Ib, followed by re-infusion of expanded MCPyV LT-ag-specific CD8+ T-cells resulted in a complete response in two of three metastatic lesions and a delayed appearance of new metastasis compared to controls [205]. Tumor necrosis factor (TNF) can be considered as an alternative for treating MCC because this cytokine displayed a high efficacy in three patients [206,207], and in one patient out of three treated with IFNγ plus TNFα a complete response was noticed, while a partial and no response was observed in two others [208]. Because the three aforementioned TNFα studies were performed before the discovery of MCPyV, the presence of virus in the tumors was not known. Interestingly, patients who have been treated with TNFα inhibitors show an increased risk of developing MCC [209,210]. Another immunotherapy approach for the treatment of MCC could be PD-1 ligand and Tim-3, with these receptors highly expressed on MCPyV-specific CD+ T-cells. Drugs targeting the PD-1/PDL-1 pathway such as nivolumab (a blocking antibody against PD-1), pembrolizumab (anti-PD-1 antibody) and BMS-936559 (anti-PDL-1 antibody) have been used in other cancers and may be used to treat MCC [211]. 

Therapeutic strategies aimed at other emerging hallmarks of cancer have been little explored. Intratumoral delivery of anti-microRNA may help in silencing viral microRNA or viral-induced cellular microRNA, while RNA interference may turn off the expression of viral oncoproteins. RNA interference targeting LT-ag has been shown to abrogate HPyV replication *in vitro* and suppress tumor growth *in vitro* and in an animal model [180,212,213,214,215,216,217]. The use of exosomes as vaccines against cancer and infectious diseases has been suggested and exosomes pulsed with MCPyV LT-ag could be considered to treat MCPyV-positive MCC patients [187,218]. Lastly, therapeutic strategies directed against the metabolic changes in tumor cells (anti-glycolysis therapy) may be considered. The hexokinase inhibitor 2-deoxyglucose inhibited growth of fibroblasts transformed by the telomerase catalytic subunit plus SV40 LT-ag, and by the telomerase catalytic subunit plus SV40 LT-ag plus st-ag [165]. To our best knowledge, the effect of 2-deoxyglucose on the growth of MCPyV-positive MCC cells has not been investigated.

## 5. Conclusion and Future Perspectives

Seroprevalence studies demonstrate that HPyV viruses are common in the human population [36]. Although HPyV LT-ag and st-ag possess proven or putative transforming properties, only MCPyV seems to be associated with human cancer. This virus encodes additional early proteins (ALTO protein and 57 kD protein), whose functions are not completely understood [4,219]. Proper immune surveillance may explain why HPyVs establish a harmless life-long infection in most individuals, while immune deficiencies may lead to viral-associated pathologies, including malignancy. The role of HPyVs in the emerging hallmarks of cancer has been little investigated and further investigations are required to elucidate the mechanisms by which HPyV-positive tumors can evade the antiviral responses of the host and affect energy homeostasis. A better understanding of the tumor microenvironment is required to comprehend the development of MCC. A possible involvement of exosomes in HPyV-induced cancer and modulation of the immune system have not been addressed, and the role of viral microRNA or HPyV-induced microRNA in tumorigenesis is incompletely understood. Unveiling the mechanisms by which these viruses participate in emerging hallmarks of cancer may therefore enable the development of novel therapeutic strategies.
